# The impact of Mirabegron on sexual function in women with idiopathic overactive bladder

**DOI:** 10.1186/s12894-019-0438-8

**Published:** 2019-01-21

**Authors:** Marilena Gubbiotti, Antonella Giannantoni, Simona Cantaluppi, Anna Chiara Coluccia, Fabio Ghezzi, Maurizio Serati

**Affiliations:** 1 Department of Surgical and Biomedical Sciences, Uro- Oncology Section, University of Perugia, S. Maria della Misericordia Hospital, 06132 Perugia, Italy; 2 Istituto Serafico di Assisi, Centro di Ricerca “InVita”, 06081 Assisi, Perugia Italy; 3 Department of Medical and Surgical Sciences and Neuroscience, Urology Section, University of Siena, Siena, 53100 Italy; 4 Department of Obstetrics and Gynecology, University of Insubria, Varese, 21100 Italy

**Keywords:** Mirabegron, Overactive bladder, Sexual dysfunction, Women

## Abstract

**Background:**

Overactive bladder (OAB) can frequently exert a negative effect on female sexual function. Mirabegron, a β3 receptor agonist, improves OAB symptoms, but there are very few information about its role on female sexual dysfunction (FSD). Aim of the study was to assess the impact of Mirabegron on FSD in women affected by OAB.

**Methods:**

Fifty sexually active women suffering from idiopathic OAB were included in the study. Patients were assessed by means of a urogynecologic physical examination and were asked to complete the 3-day voiding diary, the International Consultation on Incontinence Questionnaire- Short Form (ICIQ-SF), the Female Sexual Function Index (FSFI) questionnaire and VAS, before and 12 weeks after treatment with Mirabegron. In addition, at the same time points, patients underwent uroflowmetry with the measurement of post- void residual volume (PVR).

**Results:**

At baseline all patients were affected by OAB symptoms, with 49/50 patients (98%) presenting with FSD. At 12- weeks follow- up, OAB symptoms improved significantly in all patients, with 59.5% of subjects achieving a complete urinary continence. FSFI Total Score significantly improved in 42/50 patients (84%) from 18.9 ± 4.3 to 21.8 ± 4.5 (*p* < 0.0001). Sixteen cases (32%) presented with no FSD. Also mean ± SD scores of ICIQ-SF and VAS significantly improved (from 17.1 ± 5 to 7.9 ± 4.8 and from 3.9 ± 1.2 to 6.9 ± 1.2 respectively, *p* < 0.000).

**Conclusions:**

Mirabegron not only is able to control urinary symptoms in women with OAB, but also induces a significant improvement in their sexual life.

## Brief summary

Women with OAB may suffer from sexual dysfunction. This study shows that Mirabegron is also able to improve female sexual function.

## Background

According to the International Continence Society, Overactive Bladder (OAB) is a symptom syndrome suggestive of lower urinary tract dysfunction and includes urgency, increased day- time and night- time urinary frequency and Urge Urinary Incontinence (UUI) [1]. Prevalence of OAB has been reported to be 12.2% in women, becoming higher in patients aged > 40 years [2]. To date very few studies have investigated the impact of OAB symptoms on female sexual function [3, 4]. One of previous studies demonstrated a great deterioration in sexual Quality of Life (QoL) in women with OAB than in those with only urinary incontinence. In addition, it has been demonstrated that the improvement in OAB symptoms, as induced by a pharmacological treatment, can determine an amelioration also in female sexual life [5]. Mirabegron is a β3- agonist recently introduced in the treatment of OAB, with proven efficacy in reducing urgency and UUI, and with a good safety and tolerability profiles [6–8]. Moreover, Mirabegron has also been shown to exert a beneficial impact on health related QoL in treated patients [9]. Actually, there are no data on the impact of Mirabegron on sexual function in women affected by OAB. Aim of the study was to determine whether Mirabegron, used as a treatment for OAB symptoms, can have a positive effects also on Female Sexual Dysfunction (FSD) and to identify possible relationships between the improvement in urinary symptoms and changes in female sexual life.

## Methods

This was an observational, analytical, prospective study performed in two Italian centers. The procedures were performed in accordance with the Declaration of Helsinki and approved by the local Ethics Committee (CEAS No. 12394/18). Patients gave their informed written consent to the study. Fifty sexually active women, with a confirmed diagnosis of idiopathic OAB, attending the Urogynecology outpatient clinic were consecutively enrolled. OAB was defined using the International Continence Society definition [10]: patients having urinary symptoms of urgency with or without UUI, with frequency and nocturia. All patients reported day- time urinary frequency of 8 or more times per day, urgency that may or may not accompanies UUI, urinary symptom’s duration of 3 months or longer. Subjects were excluded from participation if they had documented recurrent urinary tract infections (UTIs ≥3 episodes/year), urinary retention or clinically relevant Post- Void Residual volume (PRV), clinically significant voiding dysfunction (symptoms of hesitancy, slow streaming, intermittency, straining to void, feeling of incomplete bladder emptying), previous anti- incontinence or prolapse surgery, presence of a neurological cause of OAB, Pelvic Organ Prolapse Quantification (POPQ ≥ stage II), any medical condition contraindicating the use of β3- agonist medications and previous pharmacological treatment or intradetrusor injections of botulinum toxin for OAB during the previous 3 months. Not sexually active women were also excluded from the study.

### Screening visit

At the screening visit, patients underwent medical history, including a detailed sexual history, urogynecologic assessment (performed with the patient in the lithotomy position, and POP classified during a maximal Valsalva manoeuvre according to the POPQ system), blood pressure measurement, urinalysis and cultures, uroflowmetry with PVR, and VAS to score the impact of urinary symptoms on QoL (0 = worse; 10 = best). A 3- day voiding diary was used to record daily urgency and UUI episodes and day- time and night- time urinary frequencies. Patients underwent a validated Italian version of “International Consultation on Incontinence Questionnaire- Short Form” (ICIQ-SF) questionnaire. ICIQ-SF is a specific and brief questionnaire: it has four questions that rapidly assess the impact of UI and qualify the urinary losses of analysed patients in terms of frequency and severity, and eight self- diagnostic items related to causes or situations of UI experienced by patients. The questionnaire has a 0–10 numerical scale for assessment of the UI impact. Zero indicates little interference of UI in the interviewer’s daily life, and 10 represents a lot of interference. The total score is obtained by summing the questions related to frequency, quantity and impact on daily life, and can vary from 0 to 21 [11].

The assessment of sexual function and sexual QoL were performed by patient- reported questionnaires [12]. All patients were asked to complete the Female Sexual Function Index (FSFI) questionnaire, by using its validated Italian version [13]. FSFI is one of the most frequently adopted questionnaires in assessing female sexual function and dysfunction, and it has been strongly recommended from the latest report of International Consultation on Sexual Medicine. [14, 15] FSFI allows to study and classify the following domains: (a) desire (b) arousal (c) lubrication (d) orgasm (e) satisfaction and (f) pain. A total FSFI score of 26.5 is the cut-off to discriminate women with and without sexual dysfunction [16].

### Treatment

All patients received a 12- week oral Mirabegron (50 mg once a day). We considered patients to be compliant only if they adhered to the therapy every day for 12 weeks.

### Follow- up

At the 12- week follow- up examination, patients underwent blood pressure measurement, the 3- day voiding diary, uroflowmetry with PVR and ICIQ-SF and FSFI questionnaires and VAS.

### Objective efficacy outcomes

Were: changes from baseline in the mean number of day- time and night- time urinary frequency, urgency and UUI; maximum flow rate at uroflowmetry with PVR.

### Subjective efficacy outcomes

Were: changes from baseline in ICIQ-SF and FSFI scores and in VAS scores.

### Side effect

Presence of side effects possibly related to the use of Mirabegron, such as hypertension, nasopharyngitis, and urinary tract infection was investigated. A daily blood pressure recording was performed during the first week of treatment and at the end of the study.

Data were prospectively collected into a specifically designed digital database.

#### Statistical analysis

Statistical analysis was performed with IBM-SPSS v.17 for Windows (IBM Corp, Armonk, NY, USA). Student’s t test and the Mann–Whitney U test were performed to compare continuous parametric and nonparametric variables, as appropriate. Continuous variables were reported as median and Interquartile Range (IQR) or mean ± SD. Relationships between differences in FSFI and ICIQ-SF, urgency episodes, UUI, and frequency, were evaluated by Pearson coefficient analysis. A *P* value less than or equal to .05 was considered significant. Power calculation were done and we estimated that with 50 participants, the study would have 80% or greater power to detect a mean difference between the pre- and the post treatment FSFI total score of at least 3 points (considered clinically relevant), assuming a standard deviation of 2 and a two-sided type I error rate of 0.05.

## Results

All the 50 patients completed the study. Mean ± SD age was 49.3 ± 11.3 years. All patients completed the questionnaires at the start of the study and at 12- week follow- up. Baseline characteristics of the study population are summarized in Table 1.

**Table 1:** Baseline characteristics of 50 women affected by idiopathic OAB with sexual dysfunction, treated with oral Mirabegron, 50 mg/day for 12 weeks

	*N* = 50
Age (years)	50 (36–60)
BMI (kg/m^2^)	23.3 (23–28)
Obese (BMI ≥ 30	5 (10%)
Menopausal	19 (38%)
Previous vaginal deliveries	1 (1–2)
Macrosome (≥4000 g)	10 (20%)
Operative delivery (vacuum/forceps)	5 (10%)

Data are expressed as absolute number (%) or median (IQR)

### Clinical results

At initial evaluation (Table 2), all patients had increased day- time urinary frequencies and all complained of urgency. Forty- seven (94%) patients presented with UUI. At the 12- week follow- up, mean ± SD day- time and night- time urinary frequencies, daily urgency and UUI episodes decreased significantly (*p* < 0.0001). Twenty- eight/fifty (56%) patients achieved a complete urinary continence. On uroflowmetry, we did not observe any significant difference in the Maximum Flow rate (Q_max_) and in PVR between the pre- treatment and post- treatment values. Mean ± SD ICIQ-SF score significantly increased, from 17.1 ± 5 (pathologic value) to 7.9 ± 4.8 (normal value; *p* < 0.0001). Mean ± SD VAS score significantly increased, from 3.9 ± 1.2 to 6.9 ± 1.2 (*p* < 0.0001).

**Table 2:** Urinary symptoms and uroflowmetry results in 50 women affected by idiopathic OAB and sexual dysfunction before and 12- weeks after treatment with oral Mirabegron, 50 mg/day

Urinary symptoms and uroflowmetry results	Baselin (mean ± SD)	12 weeks f-up (mean ± SD)	*p* value
Day-time urinary frequency/day	10.1 ± 2.2	6 ± 1.1	< 0.0001
Night-time urinary frequency/day	1.6 ± 1.2	0.7 ± 0.8	0.0001
Daily urgency episodes	5.2 ± 2.3	2.3 ± 1.8	< 0.0001
Urge urinary incontinence frequency/day	2.1 ± 1.3	0.7 ± 0.9	< 0.0001
Q_max_(ml/sec)	25.1 ± 8.3	25.2 ± 7.9	0.83
Post-void residual volume(ml)	5.8 ± 9.5	3 ± 5.8	0.32

### Sexual function and sexual QoL

At initial evaluation, 49/50 patients (98%) presented with FSD (FSFI Total Score < 26.55). At 12- weeks follow- up, 42/50 patients (84%) reported improvements in the FSFI Total Score and 16 patients (32%) had no FSD (Table 3). Mean ± SD FSFI Total Score significantly increased from 18.9 ± 4.3 to 21.9 ± 4.5 (*p* < 0.0001). The domains most relevantly impaired were arousal and lubrication, orgasm and pain. After treatment with Mirabegron, an improvement was noted in all the domains but pain remained unchanged (Table 3). At the 12- weeks follow- up, when comparing changes in sexual function between the 28 continent patients and the remaining incontinent ones, FSFI domains significantly improved in those patients who obtained a complete urinary continence. (Table 4).

**Table 3:** FSFI scores in 50 patients affected by idiopathic OAB symptoms and sexuadysfunction, before and 12 weeks after treatment with oral Mirabegron, 50 mg/day

FSFI domains	Baseline (mean ± SD)	12-weeks f-up (mean ± SD)	*p* value
Desire	3.03 ± 0.65	3.51 ± 0.88	0.04
Arousal	2.87 ± 0.61	3.35 ± 0.94	< 0.0001
Lubrication	3.31 ± 0.72	3.93 ± 1.06	0.0008
Orgasm	3.36 ± 0.74	3.80 ± 0.96	0.0001
Satisfaction	3.33 ± 0.91	4.04 ± 1.05	0.0008
Pain	3.24 ± 0.88	3.52 ± 0.93	0.58
Total FSFI score	18.9 ± 4.3	21.9 ± 4.5	< 0.0001

**Table 4:** FSFI scores in 50 women affected by idiopathic OAB symptoms and sexual dysfunction, before and 12 weeks after treatment with oral Mirabegron, 50 mg/day

Patients (No.)	FSFI Total Score baseline (mean ± SD)	FSFI Total Score 12-weeks f-up (mean ± SD)
Continent (28)	22 ± 3.7	27.5 ± 3.5
Incontinent (22)	14.1 ± 3.1	21 ± 3.2

Comparison between continent and incontinent patients before and after treatment with Mirabegron

We found a significant relationship between improvements in ICIQ-SF and FSFI scores after treatment (*r* = 0.5; *p* = 0.007). Moreover, we could detect a significant relationship between the reduction in daily urgency episodes and the improvement in FSFI score *(r* = 0.45; *p* = 0.03). On the contrary, the relationship between daily UUI episodes, urinary frequency and FSFI total score was not statistically significant (*r* = − 0.1; *p* = 0.35).

#### Side effects

None of the patients reported any increase in systolic and/or diastolic pressure along the whole follow- up. At the 12- week follow- up, 2 patients presented with UTIs.

## Discussion

This study seems to show that the treatment of OAB with Mirabegron 50 mg per day for 12 weeks is able to improve sexual function in female patients with OAB.

In the last years, an enormous interest has emerged in sexual life of patients affected by OAB. Nevertheless, there is still limited knowledge about the impact of OAB therapies on sexual function.

This study was addressed to evaluate whether treatment with the β3- agonist Mirabegron and a corresponding improvement in OAB symptoms could be associated with an amelioration on FSD. Herein we demonstrate for the first time that Mirabegron, given to control OAB in women, was able not only to improve urinary symptoms but also many aspects of sexual life of these patients in the short- term follow- up.

Specifically, we found that all OAB symptoms and ICIQ-SF total score significantly improved at the 12- week follow- up. At the same time point, a significant amelioration was found also in sexual dysfunction, as assessed by the FSFI questionnaire. In addition, the impact of urinary symptoms on QoL, as assessed by VAS, also showed a significant reduction. Worth of noting, ameliorations in urinary urgency and in ICIQ-SF scores were significantly related to the improvement in FSFI scores, with desire, arousal, satisfaction, lubrication and orgasm being the most improved domains.

Indeed, at baseline, our patients presented with FSFI scores (mean ± SD: 18.9 ± 4.3) lower than that reported in the accepted definition of FSD (FSFI ≤26.5), with arousal, lubrication, orgasm- related problems and pain most relevantly impaired. Twelve weeks after treatment with Mirabegron, FSFI total score significantly improved, particularly in those patients who achieved a complete urinary continence, although patients still presented with low FSFI values (mean ± SD: 21.9 ± 4.5), which did not reach the suggested cut- off. In the literature, the cut- off point in the FSFI score to define clinical improvement is unclear and FSFI is adopted as an assessment tool to determine any change post- treatment.

Few data exist in the literature regarding the effect of OAB treatments on female sexual function. Sand et al. demonstrated that women treated with transdermal oxybutynin reported improvements in sexual function at 6 months [17]. In the study of Hajebrahimi et al., 30 sexually active OAB women underwent treatment with tolterodine immediate release in order to control their OAB symptoms. In these patients, significant improvements have been reported not only in urinary symptoms but also in the mean ASEX score at all the considered follow- up time points [18]. In a randomized, placebo- controlled study, Rogers and co-workers observed a significant amelioration in urinary symptoms and sexual dysfunction in female patients treated with tolterodine extended- release, as compared to those treated with placebo, at 3 months follow up [19]. Finally, significant improvements in total FSFI score and in OAB symptoms have been observed also in patients undergoing sacral neuromodulation or onabotulinumtoxin A intradetrusor injections [5, 20]. Although Mirabegron has been demonstrated to be effective and safe in the treatment of OAB in naïve patients or in those refractory to anticholinergics [21], to date data related to an eventual effect on sexual dysfunction are completely lacking. The results in our study may be in favour of a causal relationship between urinary symptoms and sexual dysfunction in women [22]. Since Mirabegron induced improvements in all OAB symptoms and a complete urinary continence in a large proportion of patients, we retain that the positive effect on sexual dysfunction could be attributed to an indirect effect by the urologic clinical and QoL amelioration. Overall, Mirabegron was well tolerated without any systemic side effects along the whole follow- up and it did not affect the bladder emptying, as already demonstrated in the literature.

Possible limitations of the present study are the short-term follow- up and the lack of a control or a placebo group. Indeed, no patient was lost to follow- up, and the limited improvement in sexual function observed in patients not reaching a complete urinary continence can strengthen these results.

## Conclusion

This study could advance the understanding of FSD in women with OAB and the possibility of intervening with new pharmacologic oral agents in the treatment of OAB. In our study the improvement in sexual dysfunction observed in patients treated with Mirabegron can be explained by the amelioration in OAB symptoms and in urinary incontinence. However, future trials with a larger patient population should confirm these preliminary findings. In conclusion, according to our research, treatment of OAB with Mirabegron 50 mg per day for 12 weeks is able to improve sexual function in women affected by OAB. This finding can improve our counseling when we prescribe this pharmacological therapy of OAB.

## Abbreviations

BMI: Body Mass Index
FSD: Female Sexual Dysfunction
FSFI: Female Sexual Function Index
ICIQ-SF: International Consultation on Incontinence Questionnaire- Short Form
IQR: Interquartile Range
OAB: Overactive Bladder
POP(Q): Pelvic Organ Prolapse (Quantification)
PVR: Post- Void Residual volume
Q_max_: Maximum Flow rate
QoL: Quality of Life
SD: Standard Deviation
UTIs: Urinary Tract Infections
UUI: Urge Urinary Incontinence
